# Effects of prenatal childbirth education for partners of pregnant women on paternal postnatal mental health: a systematic review and meta-analysis protocol

**DOI:** 10.1186/s13643-016-0199-3

**Published:** 2016-02-03

**Authors:** Maiko Suto, Kenji Takehara, Yumina Yamane, Erika Ota

**Affiliations:** Tsuda College, 2-1-1 Tsuda-machi, Kodaira-shi, Tokyo 187-8577 Japan; National Center for Child Health and Development, 10-1 Okura 2-chome, Setagaya, Tokyo 157-8535 Japan; Ome, Tokyo Japan

**Keywords:** Antenatal education, Childbirth education, Fathers, Partners of pregnant women, Postnatal depression

## Abstract

**Background:**

The prevalence of paternal depression in the postnatal period is estimated to be approximately 10 %. Effective partner education during pregnancy has the possibility to prevent postnatal mental health problems and support expectant fathers in their transition to parenthood. This paper describes the protocol of a systematic review that will investigate the effects of prenatal childbirth education for partners of pregnant women particularly on paternal postnatal mental health.

**Methods/design:**

We will search the databases of MEDLINE, CINAHL, EMBASE, PsycINFO, ERIC, and CENTRAL, using related search terms such as “partners of pregnant women,” “education,” and “prenatal support.” Searches will be limited to randomized trials. Two review authors will independently screen eligible studies and assess risk of bias. We will report structured summaries of the included studies and conduct meta-analysis.

**Discussion:**

Postnatal mental health of fathers is reported to have various effects on the health of the whole family. Therefore, support for expectant fathers is an important issue in the maternal and perinatal health-care system. However, resources on prenatal childbirth education for partners of pregnant women remain limited. The results of this review will provide evidence for prenatal education programs for expectant fathers.

**Systematic review registration:**

PROSPERO CRD42015017919

**Electronic supplementary material:**

The online version of this article (doi:10.1186/s13643-016-0199-3) contains supplementary material, which is available to authorized users.

## Background

The majority of prenatal childbirth education programs are targeted at pregnant women. However, in recent years, men are expected to play a more active role in childbirth and childcare, and the number of educational programs that focuses on partners of pregnant women has increased [1].

Several studies have reported the prevalence of postpartum depression among fathers to be approximately 10 % [2–4]. Paternal postpartum depression may not only affect the mental health of fathers themselves but can also have an impact on their partners and children. Some studies have reported that paternal depression was associated with the risk of maternal depression and poor marital relationships [3, 5]. Paternal postpartum depression is also associated with impaired parenting practices and can lead to negative child development [2, 6–8]. Therefore, support for expectant fathers is a very important issue in maternal and perinatal healthcare.

Compared to women, it can be more difficult for men to obtain information about childbirth and childcare. Attendance at prenatal childbirth education classes can provide a positive influence on men’s postnatal mental health. Although educational programs on the role of fathers in childbirth and child-rearing have become more widely available, it remains uncertain whether prenatal childbirth education for partners of pregnant women is effective for improving paternal postnatal mental health. The results of this review will provide much-needed evidence for prenatal education programs targeting expectant fathers.

## Methods/design

### Objectives

The objective of this study is to examine the effectiveness of prenatal childbirth education for partners of pregnant women in improving paternal postnatal mental health.

### Type of studies

We will include randomized control trials (RCTs) and cluster RCTs. If there are less than three eligible studies, we will include quasi-RCTs (quasi-random method of allocation such as alternation, date of birth, or medical record number). We will exclude non-randomized controlled before-after studies, where random allocation or some quasi-random method of allocation was not used. We will follow the Cochrane definitions and criteria for RCTs and controlled clinical trials [9].

This protocol is registered with PROSPERO (International prospective register of systematic reviews) at the National Institute for Health Research and the Centre for Reviews and Dissemination (CRD) at the University of York (registration number: CRD42015017919). This protocol is reported according to the Preferred Reporting Items for Systematic review and Meta-Analysis Protocols (PRISMA-P) 2015 checklist in Additional file 1 [10].

### Type of participants

The participants are men who are partners of pregnant women.

### Type of interventions

We will assess childbirth education provided for partners of pregnant women in the prenatal period. We will include educational programs involving both pregnant women and their partners. Educational interventions provided only for pregnant women will be excluded. Any type of education such as clinical-based, home-based, internet-based, telephone-based, pamphlet-based, and both individual and group educational programs will be included. We will not limit studies by number or timing of prenatal education classes.

The control intervention will be either no educational program or other types of programs that are usual in the prenatal period.

### Type of outcome measures

#### Primary outcomes

1. Paternal depression measured using a valid assessment tool, such as the Edinburgh Postnatal Depression Scale (EPDS), the Center for Epidemiologic Studies Depression Scale (CES-D and CESD-R), the Beck Depression Inventory (BDI), or the General Health Questionnaire (GHQ).
2. Satisfaction with the postnatal couple relationship measured using a valid assessment tool defined by the study authors.

#### Secondary outcomes

1. Partners’ parenting behavior, distress, and parent-infant interaction measured using a valid assessment tool defined by the study authors.
2. Partner attendance at birth and satisfaction of childbirth.
3. Partners’ fears or anxiety about childbirth measured using a valid assessment tool defined by the study authors.
4. Satisfaction with prenatal childbirth education measured using a valid assessment tool defined by the study authors.
5. Maternal depression measured using a valid assessment tool such as the EPDS, CES-D (CESD-R), BDI, or GHQ.
6. Child outcomes such as emotional and behavioral development, psychiatric disorders, or difficult temperament measured using a valid assessment tool defined by the study authors.

If the same outcomes are measured at various assessment points, we will conduct subgroup analysis according to time after birth, such as 1 month or 1 year after birth.

### Electronic searches

We will conduct this systematic review in accordance with the PRISMA statement [11]. We will search the following databases: MEDLINE, CINAHL, EMBASE, PsycINFO, ERIC, and CENTRAL using search terms related to partners of pregnant women, education, and prenatal support. We will include all languages in our searches. We will not limit the publication date. Searches will be limited to randomized trials. Our search strategy will be assessed by an experienced information specialist of the National Center for Child Health and Development. The search strategy is shown in more detail in Additional file 2.

### Data collection and analysis

#### Inclusion criteria

1. Participants: partners of pregnant women. We will not limit participants’ characteristics such as socio-demographics or parity (of pregnant women). We will include unmarried couples.
2. Study design: RCTs (including cluster RCTs and quasi-RCTs).
3. Intervention: education program for partners of pregnant women to improve paternal mental health. We will include studies where the intervention was delivered to both the pregnant woman and the partner.
4. Intervention setting: clinical-based, internet-based, telephone-based, and both individual and group educational programs. We will include any type of education.
5. Time of assessment: we will set a time-limit on the outcome within 12 months after birth.

#### Exclusion criteria

1. Excluded studies: descriptive studies, controlled before-after studies.
2. Excluded intervention programs:
  - Programs provided only for pregnant women
  - Programs targeted exclusively at smoking, alcohol, HIV, breastfeeding, or specific childbirth outcomes such as cesarean or epidural rates
3. Excluded participants: participants with any serious physical or mental illness
4. Excluded publications: non-academic journals and reports

### Data extraction and management

Two review authors (MS and YY) will independently screen the titles and abstracts of studies that meet the search strategy in order to identify eligible studies for inclusion. Full texts of eligible studies will be obtained. The same two review authors will then independently screen the full text and judge whether the studies should be included or excluded using the same criteria applied at initial screening. Any disagreements will be resolved through discussion or consultation with other authors (EO and KT).

### Assessment of risk of bias in included studies

Two review authors (MS and YY) will assess risk of bias independently in accordance with the Cochrane Handbook for Systematic Reviews of Interventions [9]. We will use the following characteristics to assess the risk of bias: selection bias, performance bias, attrition bias, reporting bias, and other bias.

Further, if possible, we will carry out statistical meta-analysis. MS and YY will assess independently whether or not to include studies in the meta-analysis. Any disagreement between the two review authors will be resolved through consulting with other authors (EO and KT).

### Measures of treatment effect

If same type of interventions are used and outcomes are measured using the same tools between trials, we will calculate the mean difference and 95 % confidence intervals for continuous outcomes and present the results of the summary risk ratio and 95 % confidence intervals for dichotomous outcomes.

If it is difficult to conduct a quantitative synthesis or meta-analysis because of the range of different population, interventions, or outcomes, we will report narrative summaries of the included studies about population characteristics, type of interventions, type of outcomes, and intervention effects.

### Dealing with missing data

For included studies, we will assess the level of missing data in the primary outcome by using sensitivity analysis. Statistical analyses will be conducted based on intention-to-treat analysis as much as possible.

### Assessment of heterogeneity

We will assess heterogeneity by using chi-squared and *I*-squared statistics. We will consider that heterogeneity exists if chi-squared value is lower than 0.10 and *I*-squared value is greater than 50 %.

### Assessment of reporting bias

If there are 10 or more studies in the meta-analysis, we will investigate reporting biases using funnel plots and visually interpret for the funnel plot asymmetry.

### Data synthesis

If possible, we will carry out statistical meta-analysis using Review Manager Version 5.3 (Cochrane Collaboration software). If we are not able to analyze data due to a lack of data or high heterogeneity, we will report the results narratively.

### Subgroup analysis and investigation of heterogeneity

If the necessary data are available, we will implement subgroup analysis for primary outcomes of the following:

1. Intervention setting: e.g., clinical-based, internet-based versus telephone-based
2. Size and participants of class:
  - Individual versus group
  - Both parents versus only partners of pregnant women
3. Characteristics of partners of pregnant women:
  - Socio-demographic factors, e.g., age
  - Partners of pregnant women with previous children versus without previous children
4. Timing of intervention: first, second versus third trimester of pregnancy
5. Number of childbirth education classes: partners of pregnant women who only once participated in a prenatal childbirth education class versus those who participated more than twice
6. Place of studies:
  - Countries of studies: low- and middle-income countries versus high-income countries (defined by World Bank criteria) [12]
  - Regions of studies: Africa, America, Asia, Europe, versus Oceania (defined by United Nations Statistics Division) [13]

### Sensitivity analysis

We will conduct sensitivity analysis, excluding studies with a high risk of allocation concealment or incomplete outcome data, if included studies are judged to have a high risk of bias for the primary outcomes.

### Summary of findings

We will use the Grading of Recommendations, Assessment, Development and Evaluation (GRADE) approach to assess the quality of the evidence across studies [14]. The quality of the evidence for each outcome will be assessed by study limitation, inconsistency, imprecision, indirectness, and publication bias, and the quality will be judged as high, moderate, low, or very low. We will present our results with a GRADE summary table [15].

## Discussion

The effectiveness of the current prenatal education programs for expectant fathers on paternal postnatal mental health is still uncertain. This review and meta-analysis will provide evidence on the effectiveness of such programs on paternal postnatal mental health. The results of this review will help to develop evidence-based prenatal education programs for expectant fathers.

## Additional files

Additional file 1:
**The Preferred Reporting Items for Systematic Reviews and Meta-Analyses for Protocols 2015 (PRISMA-P 2015) checklist was used in this protocol.** (DOC 80 kb) Additional file 2:
**The search terms and strategies that will be used to identify eligible studies.** (DOCX 16 kb)

## Abbreviations

BDI: Beck Depression Inventory
CES-D: Center for Epidemiologic Studies Depression Scale
EPDS: Edinburgh Postnatal Depression Scale
GHQ: General Health Questionnaire
GRADE: Grading of Recommendations, Assessment, Development and Evaluation
RCTs: randomized control trials
