# Supplementary material for: Effects of prenatal childbirth education for partners of pregnant women on paternal postnatal mental health: a systematic review and meta-analysis protocol
Source: Syst Rev. 2016 Feb 3;5:21. doi: 10.1186/s13643-016-0199-3 (PMC4741014; doi:10.1186/s13643-016-0199-3)
Supplement: Additional file 2: — The search terms and strategies that will be used to identify eligible studies. (DOCX 16 kb) [file 13643_2016_199_MOESM2_ESM.docx]

**Additional file 2: Search terms and strategies**

Database Field Guide Ovid MEDLINE(R) In-Process & Other Non-Indexed Citations February 20, 2015,

Database Field Guide Ovid MEDLINE(R) 1946 to Present with Daily Update

1. Parturition/
2. parturition*.tw.
3. childbirth*.tw.
4. antenatal*.tw.
5. Prenatal Care/
6. prenatal*.tw.
7. Pregnancy/
8. pregnan*.tw.
9. or/1-8
10. Education/
11. education*.tw.
12. intervention*.tw.
13. program*.tw.
14. information*.tw.
15. resource*.tw.
16. support*.tw.
17. (care* or caring).tw.
18. or/10-17
19. 9 and 18
20. Fathers/
21. father*.tw.
22. paternal*.tw.
23. Spouses/
24. spouse*.tw.
25. husband*.tw.
26. partner*.tw.
27. or/20-26
28. 19 and 27
29. randomized controlled trial.pt.
30. controlled clinical trial.pt.
31. randomi?ed.ab.
32. placebo.ab.
33. clinical trials as topic.sh.
34. randomly.ab.
35. trial.ti.
36. groups.ab.
37. or/29-36
38. 28 and 37
39. Animals/
40. Humans/
41. 39 not 40
42. Parturition/
43. parturition*.tw.
44. childbirth*.tw.
45. antenatal*.tw.
46. Prenatal Care/
47. prenatal*.tw.
48. Pregnancy/
49. pregnan*.tw.
50. or/42-49
51. Education/
52. education*.tw.
53. intervention*.tw.
54. program*.tw.
55. information*.tw.
56. resource*.tw.
57. support*.tw.
58. (care* or caring).tw.
59. or/51-58
60. 50 and 59
61. Fathers/
62. father*.tw.
63. paternal*.tw.
64. Spouses/
65. spouse*.tw.
66. husband*.tw.
67. partner*.tw.
68. or/61-67
69. 60 and 68
70. randomized controlled trial.pt.
71. controlled clinical trial.pt.
72. randomi?ed.ab.
73. placebo.ab.
74. clinical trials as topic.sh.
75. randomly.ab.
76. trial.ti.
77. groups.ab.
78. or/70-77
79. 69 and 78
80. Animals/
81. Humans/
82. 80 not 81
83. 79 not 82
84. remove duplicates from 83
